# Adapting strategies for effective and efficient pediatric HIV case finding in low prevalence countries: risk screening tool for testing children presenting at high-risk entry points in Ethiopia

**DOI:** 10.1186/s12879-022-07460-w

**Published:** 2022-05-20

**Authors:** Wondimu Teferi, Steve Gutreuter, Alemayehu Bekele, Jelaludin Ahmed, Jemal Ayalew, Jessica Gross, Hanna Kumsa, Tenagnework Antefe, Semegnew Mengistu, Kelsey Mirkovic, Eric J. Dziuban, Christine Ross, Zena Belay, Tsegaye Tilahun, Desta Kassa, Susan Hrapcak

**Affiliations:** 1 Centers for Disease Control and Prevention, US Embassy Entoto Road, P.O.B 1014, Addis Ababa, Ethiopia; 2 Centers for Disease Control and Prevention, Atlanta, Georgia, USA; 3 Ethiopia Public Health Association, Addis Ababa, Ethiopia; 4 Addis Ababa City Administration Health Bureau, Addis Ababa, Ethiopia; 5 Amhara Regional Health Bureau, Amhara, Ethiopia; 6 United States Agency for International Development, Addis Ababa, Ethiopia; 7 Ethiopian Public Health Institute, Addis Ababa, Ethiopia

**Keywords:** Children, Risk screening, HIV, Testing, Entry point

## Abstract

**Background:**

Implementing effective and efficient case-finding strategies is crucial to increasing pediatric antiretroviral therapy coverage. In Ethiopia, universal HIV testing is conducted for children presenting at high-risk entry points including malnutrition treatment, inpatient wards, tuberculosis (TB) clinics, index testing for children of positive adults, and referral of orphans and vulnerable children (OVC); however, low positivity rates observed at inpatient, malnutrition and OVC entry points warrant re-assessing current case-finding strategies. The aim of this study is to develop HIV risk screening tool applicable for testing children presenting at inpatient, malnutrition and OVC entry points in low-HIV prevalence settings.

**Methods:**

The study was conducted from May 2017–March 2018 at 29 public health facilities in Amhara and Addis Ababa regions of Ethiopia. All children 2–14 years presenting to five high-risk entry points including malnutrition treatment, inpatient wards, tuberculosis (TB) clinics, index testing for children of positive adults, and referral of orphans and vulnerable children (OVC) were enrolled after consent. Data were collected from registers, medical records, and caregiver interviews. Screening tools were constructed using predictors of HIV positivity as screening items by applying both logistic regression and an unweighted method. Sensitivity, specificity and number needed to test (NNT) to identify one new child living with HIV (CLHIV) were estimated for each tool.

**Results:**

The screening tools had similar sensitivity of 95%. However, the specificities of tools produced by logistic regression methods (61.4 and 65.6%) which are practically applicable were higher than those achieved by the unweighted method (53.6). Applying these tools could result in 58‒63% reduction in the NNT compared to universal testing approach while maintaining the overall number of CLHIV identified.

**Conclusion:**

The screening tools developed using logistic regression method could significantly improve HIV testing efficiency among children presenting to malnutrition, inpatient, and OVC entry points in Ethiopia while maintaining case identification. These tools are simplified to practically implement and can potentially be validated for use at various entry points. HIV programs in low-prevalence countries can also further investigate and optimize these tools in their settings.

## Background

Worldwide, 1.7 million children were living with HIV in 2020, yet only half (54%) were receiving antiretroviral treatment (ART), compared to 74% of adults with HIV, despite notable expansion of pediatric ART [1]. Untreated children living with HIV (CLHIV) may suffer from severe co-morbidities, and half will not survive past their second birthday, especially perinatally-infected children [2, 3]. Early identification of CLHIV is essential to prevent HIV-related morbidity and mortality [4]. Although strategies for early infant diagnosis are in place globally, lack of timely maternal testing during pregnancy and low testing coverage throughout infancy has resulted in undiagnosed CLHIV, who may later present at health facilities for a variety of health-related services [5].

To improve pediatric ART coverage, it is crucial that HIV programs implement effective testing strategies and address barriers in identifying undiagnosed children [6]. While the World Health Organization (WHO) guidelines recommend offering testing all children with an unknown HIV status presenting to TB, malnutrition, inpatient, outpatient, or immunization entry points in high HIV burden settings, testing recommendations for low HIV burden settings are limited to children with signs, symptoms, or medical conditions indicative of HIV or HIV exposure [7–10]. Studies in high-prevalence settings demonstrated that routine opt-out testing could be an effective strategy for identifying CLHIV at outpatient department (OPD) and immunization clinics [11, 12]. High positivity rates were also found among routinely tested sick children in inpatient wards and malnutrition clinics in high-prevalence sub-Saharan African countries [13–15]. Similarly, parentless adolescents were identified as more likely to be HIV-positive [16]. However, universal testing at some service delivery points may be less efficient in countries with low HIV prevalence. Moreover, it may increase healthcare workers’ workload and compromise testing children with high risk of HIV infection, particularly in situations with limited commodities (e.g. shortages of HIV rapid test kits). Therefore, it is also important to identify approaches that improve the efficiency of testing, as measured by the average number needed to test to identify a single new CLHIV (NNT), while maintaining overall case identification. Developing risk screening tools for testing children at these entry points will be crucial to reduce unnecessary testing in low prevalence countries.

In Ethiopia, children under 15 years of age constitute over 40% of the predominantly rural population [17]. According to the recently conducted population-based HIV Impact Assessment (EPHIA) survey in urban Ethiopia, HIV prevalence among children < 15 years was 0.3%, which indicates lower national prevalence as rural areas are expected to have much lower HIV prevalence in Ethiopia [18]. However, only 40% of the estimated 44,000 CLHIV in Ethiopia were receiving ART in 2020 [19].

Current national policies in Ethiopia recommend HIV testing for all biological children of adults living with HIV (index testing), orphaned and vulnerable children (OVC) due to HIV/AIDS, children with tuberculosis (TB), and children presenting with clinical signs and symptoms of HIV/AIDS visiting health facilities at OPD and inpatient wards [20]. However, program data from the U.S. President’s Emergency Plan for AIDS Relief (PEPFAR) consistently shows significantly low positivity at some pediatric service points where routine testing is practiced, indicating a need to consider more targeted testing approaches. While evidence from high-prevalence sub-Saharan countries indicated the use of HIV risk screening tools at pediatric OPDs improves testing efficiency [21], the need for further studies to develop a more tailored approach for low-prevalence settings will be important. Furthermore, pediatric HIV risk screening tools need to demonstrate high sensitivity to reduce missed opportunities for initiation of HIV-positive children on treatment. Though programmatic screening tools adopted recently are being implemented in Ethiopia at adult and pediatric OPDs, there is little published information regarding their performance in improving the effectiveness and efficiency of pediatric case-finding.

In this study, we used facility-based data from high-risk entry points including malnutrition treatment, inpatient wards, tuberculosis (TB) clinics, index testing for children of positive adults (maternal HIV positive status or Paternal HIV positive status with unknow maternal status), and referral of orphans and vulnerable children (OVC) to develop and compare performance of potential HIV risk screening tools that improve testing efficiency and maintain case identification.

## Methods

### Study design

This cross-sectional study was conducted at 29 purposefully selected public health facilities providing high-volume HIV services in Amhara and Addis Ababa regions of Ethiopia from May 2017–March 2018. These geographic regions were selected because they performed more HIV tests compared to other regions and adult HIV prevalence is higher than the national average (but still below 5%) [22]. Descriptions of HIV testing positivity by entry point and predictors of positivity were published previously [23]. In this manuscript, we used the same dataset to develop HIV risk screening tools that can be applicable at selected entry points with lower positivity (e.g. inpatient, 0.7%, malnutrition, 0.8%, and OVC, 0.3%) [23].

### Study population and procedures

All children aged 2–14 years accompanied by a caregiver and presenting to five high-risk entry points were eligible for inclusion in the study and enrolled after written consent was obtained from caregivers, as age of consent for HIV testing in Ethiopia is 15 years. Assent was obtained from adolescents ≥ 12 years of age per the national ethics guideline [24]. OVC program rosters were used to identify children for the study based on systematic sampling method. Since it is not always easy to obtain information on parental HIV status for orphaned and vulnerable children, all OVC selected to be included in the study were referred for HIV testing regardless of their maternal HIV status. Detailed methodology has been previously described [23]. All children with unknown HIV status were offered HIV testing as part of standard of care, following the national laboratory testing algorithm. The minimum sample size was determined and allocated among the five entry points by considering client load and HIV prevalence by entry point from program data and published literature [25]. All eligible children and adolescents who visited the selected health facilities during the study period were eligible for enrollment.

### Data collection

Nutritional parameters (weight, height, and mid-upper arm circumference) and HIV test results were abstracted from the registers and charts. If anthropometric parameters were not available, they were measured by trained study staff.  Weight for height or MUAC were used to determine nutritional status as moderate or severe per the national nutrition guideline [26]. All caregivers of participants were interviewed to respond to screening questions similar to those validated by Bandason et. al in 2016 [27] plus information on school attendance, as HIV-positive children were likely to miss school [28]. Information on school attendance was recorded as not applicable where the child is not yet in school due to age or inaccessibility.

1. Has the child ever been admitted to the hospital before?
2. Has the child had poor health in the past three months?
3. Has the child been hospitalized in the past three months?
4. Is the mother of the child deceased?
5. Is the father of the child deceased?
6. Does the child have recurrent skin problems?
7. Has the child missed a lot of school due to poor health?

All data were collected using Android-based tablets using Open Data Kit (ODK).

### Data analysis

The first step in the analysis was to identify those questions which contributed to prediction of testing positive. The potential predictors of HIV positivity among newly tested children were binary indicators (1 if affirmative/present and 0 otherwise) for the presence of the following 11 presumptive risk conditions: presenting to either an index-testing or TB clinic entry point, deceased mother, deceased father, presence of recurrent skin problems, poor health during the past three months, severe malnutrition, missed school due to poor health, urban residence, presented with a primary caregiver other than a mother or father, ever hospitalized, and hospitalized during the past 3 months. Index testing as an entry point was used as a proxy for HIV-positive maternal status. Among those, plausible predictors of HIV positivity were identified by fitting bivariate logistic regression models to age, sex and the screening variables, and retaining those variables significant at P < 0.3. Those variables were used to construct the multivariable logistic screening model. The screening-model predictors were selected using *L*1 regularization [29] as implemented in the glmpath R package [30]. Regularization performs automatic variable selection by shrinking unimportant model coefficients to zero, and the glmpath implementation also imposes as small, fixed penalty on the *L*2-norm which provides robustness in the presence of collinearity. The best model was selected from the regularization path of models based on Akaike’s Information Criterion (AIC). Regularization identifies the data-generating variables with high probability, but the coefficients are biased toward zero. Therefore the final logistic screening model was obtained by maximum-likelihood estimation using the predictors identified by regularization [29]. The screening tools were based either on the covariates in the final multivariable logistic screening model or the parameter estimates from that model.

The first screening tool, referred to as the “*Z-HRST*” (Zimbabwe HIV Risk Screening Tool), was based on the unweighted method of Bandason et al. [27] using the covariates remaining in the final logistic screening model. Each covariate was assumed to contain equal risk of HIV positivity, and the screening score is the sum of the number of affirmative responses for each child, which ranges from 1–6. The performance of this first screening tool was based on overly optimistic in-sample estimates of sensitivity and specificity. The second and third tools were based on tenfold cross-validation [31, 32] of the final logistic screening model to produce out-of-sample estimates of performance, which approximates the performance in new data. For the second tool, referred to as “*exact logistic*”, the scores were the cross-validated predicted probabilities of HIV positivity from the logistic screening model. Implementation of that approach would require the use of electronic devices to compute those probabilities. The third method (“*rescaled logistic*”) eliminates the technological requirement by rescaling the logistic model coefficients for the predictor covariates to the closed interval of 1–4, then rounding each to the nearest whole number (i.e. affirmative responses to the screening questions will have values from 1–4 and negative responses contribute a value of 0). The screening score for each child is the sum of those values.

Receiver-operating characteristic (ROC) curves were developed for each screening tool, and screening threshold scores for each were based on the combination of sensitivity and specificity. We considered only those screening thresholds that yielded sensitivity greater than 90% to ensure future testing of the vast majority of undiagnosed CLHIV. ROC curves and 95% bootstrap confidence intervals (CI) for sensitivity and specificity at the threshold scores for each of the screening tools were then computed. All computations were performed using the screenr [33] package for R [34], which integrates and simplifies use of the glmpath [30] and pROC packages [35].

## Results

A total of 2112 children with previously unknown status were enrolled and tested for HIV with consent during the study period. The median age of participants was 8 years (1st and 3rd quartiles; 4 and 11, respectively). The majority (85.7%) of care givers were parents. Nearly a third of the children enrolled in the study were not yet attending school either due to younger age or inaccessibility. HIV positivity across all types of entry points was 1.9% (95% CI: 1.4–2.6%) and ranged from a high of 8.2% (95% CI: 5.6–11.8%) in index testing to a low of 0.3% (95% CI: 0.1–1.1%) in OVC (Table 1). HIV positivity was lower for malnutrition (0.8%) and inpatient (0.7%) entry points compared to index and TB (1.8%). The majority (n = 28/40, 70%) of CLHIV were identified through index testing. Overall, an average of 53 tests would be required to identify one CLHIV with higher numbers needed in OVC (NNT 333), inpatient (NNT 143) and malnutrition (NNT 125) entry points in the absence of screening.

**Table 1:** Enrollment in the study and HIV positivity of 2–14-year-old children at clinical entry points

Entry point	Number of participants enrolled	HIV positive	Percent positive (95% CI)	Number needed to test (NNT) to Identify 1 New CLHIV
Index testing	340	28	8.2 (5.8–11.6)	12
TB clinic	166	3	1.8 (0.6–5.2)	55
Malnutrition site	598	5	0.8 (0.4–1.9)	120
Inpatient ward	300	2	0.7 (0.2–2.4)	150
OVC testing	708	2	0.3 (0.1–1.0)	354
Total	2112	40	1.9 (1.4–2.6)	53

The final logistic screening model—which produced both the smallest AIC and largest partial area under that portion of the ROC curve for which sensitivity was at least 80%—retained six predictors of HIV positivity (recurrent skin problems, enrolled from index testing or TB clinic, mother deceased, severe malnutrition, urban residence and missed school due to poor health), provided the basis for screening tools (Table 2). The presence of recurrent abnormal skin conditions had the strongest association with test positivity (aOR = 15.7 (95% CI: 7.5–33.3), followed by entry through index testing or TB clinic (aOR = 12.0 (95% CI: 5.6–28.5).

**Table 2:** Predictors of HIV positivity among 2–14 year-old children in Ethiopia from multivariable logistic model obtained from *L*1 regularization

	Coefficients		Rescaled^b^ Coefficients	Adjusted^c^ odds ratios (95% CI)
Screening predictor	Regularized	MLE^a^		
Recurrent skin problems	2.74	2.76 (2.02– 3.51)	4	15.7 (7.5–33.3)
Enrolled from index testing or TB clinic	2.46	2.49 (1.72–3.35)	4	12.0 (5.6–28.5)
Mother deceased	1.46	1.50 (0.15–2.70)	2	4.5 (1.2–14.8)
Severe malnutrition	1.46	1.49 (0.69– 2.28)	2	4.4 (2.0– 9.7)
Urban residence	1.17	1.21 (0.20–2.40)	1	3.3 (1.2–11.1)
Missed school due to poor health	0.75	0.77 (-0.15–1.62)	1	2.2 (0.9–5.0)

^a^Maximum-likelihood estimates

^b^MLE coefficients rescaled to whole numbers in (1,4)

^c^Adjusted for effects of the other covariates

**Fig. 1:**
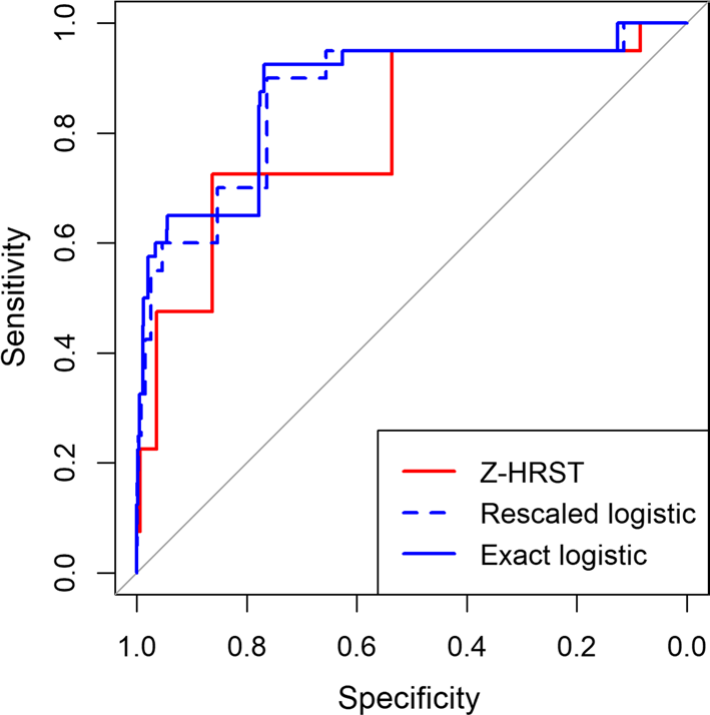
Receiver-operating characteristic (ROC) curves for the three candidate screening tools. In-sample curves are shown for the logistic tools for comparison of the overly optimistic Z-HRST tool

The three screening tools resulted in different ROC curves (Fig. 1). Both screening tools based on the cross-validated final logistic regression model had larger standardized partial areas [35] under portions of their ROC curves for which sensitivity ≥ 80% than those based on the Z-HRST method (Table 3).

**Table 3:** Performance characteristics of four candidate tools for screening 2–14 year-old children for HIV testing in Ethiopia^a^

Screening tool	pAUC	Threshold score^b^	Sensitivity (95% CI)	Specificity (95% CI)	NNT
Z-HRST	0.76	2	95.0 (87.5–100)	53.6 (51.5–55.7)	26.3
Exact logistic	0.77	0.0047	95.0 (87.5–100)	61.4 (59.4–63.6)	22.0
Rescaled logistic	0.78	4	95.0 (87.5–100)	65.6 (63.5–67.6)	19.7

^a^Out-of-sample performance of the logistic tools is based on tenfold cross-validation, whereas the value of the partial area under the receiver operating characteristic curve (pAUC) for sensitivity ≥ 80%, sensitivity, specificity and the average number of tests required to find a single HIV-positive child (NNT) is overly optimistic for the Z-HRST (Zimbabwe High-Risk Screening Tool) method

^b^Sums of affirmative responses for the Z-HRST tool, estimated probability of testing positive for the exact logistic tool, and the sum of the rounded rescaled logistic parameter estimates for the rescaled logistic tool

The screening tool based on the Z-HRST method, where the eligibility score for testing is 2, produced an in-sample specificity of 53.6% with sensitivity of 95%, requiring an average of 26 tests to identify a single CLHIV (Table 3). This tool risks missing a child with maternal HIV positive status or presenting to TB entry point from testing in the absence of affirmative response for any other screening item.

The screening tool based on exact logistic approach produced an out-of-sample sensitivity of 95% (95% CI: 87.5–100%) and specificity of 61.4% (95% CI: 59.4–63.6%). By applying this tool, the NNT can be reduced to as few as 22 children.

The screening tool based on the rounded rescaled logistic method using threshold of 4 also achieved the same out-of-sample sensitivity as the exact approach, but produced an out-of-sample specificity of 65.6% (95% CI: 63.5–67.6%), and the NNT is 19.7. This tool would also decrease the NNT in malnutrition (NNT 125 to 46), inpatient (NNT 143 to 52), and OVC (NNT 333 to 121) entry points.

## Discussion

Expanding services for prevention of mother-to-child transmission has resulted in steep declines in new pediatric HIV infections [36], making it essential to revise strategies for testing children visiting health facilities in resource-limited settings. Optimizing testing is necessary to identify more CLHIV and increase coverage for pediatric ART. As programs evolve, the balance between continuing to identify a high number of CLHIV and improving testing efficiency becomes increasingly important to maximize use of human resources and commodities, especially in the context of funding constraints. Reducing the volume of unnecessary pediatric HIV testing increases availability of HIV test kits for high-risk children, increases testing efficiency, and identifies undiagnosed CLHIV. In this study, we aimed to provide insight for policy makers and implementers in low-prevalence countries to continually review and explore their case-finding strategies.

The high proportion of CLHIV identified from index testing (70%) reinforces current recommendations to enhance universal HIV testing coverage among biological children of adults living with HIV [37] and explore innovative interventions to avoid missed opportunities at this entry point. Two of the three screening tools developed in this study are potentially applicable as they guarantee that all children presenting to the index testing or TB entry point would be automatically eligible for HIV testing. Moreover, these screening tools will reduce the NNT by up to 62%, which would significantly reduce unnecessary HIV testing. Although all three screening tools achieved a sensitivity above 90%, those based on the logistic model had better specificity.

There have been several studies evaluating pediatric risk screening tools in Asia and southern African countries. A meta-analysis by Clemens et al. [21] of seven studies that evaluated pediatric HIV risk screening tools for outpatient and inpatient settings showed sensitivity ranged from 71 to 96% and specificity ranged from 25 to 99%. The two screening tools based on logistic analysis in this study produced more effective combinations of sensitivity and specificity than all but one of the studies reviewed by Clemens et al. The only previous tool that performed better was based on clinical examinations by attending physicians and included measures that cannot be provided by caregivers [38], making it more difficult to use in rural settings. Our tools achieved high sensitivity capable of screening in most CLHIV, yet even the lowest performing screening tool in this study can reduce the NNT by half compared to universal testing in inpatient, malnutrition, and OVC entry points.

In addition to identifying the highest performing screening tools, methods for operationalization of screening tools need to be considered. The Z-HRST screening tool is attractive because implementation consists of simply recording the number of affirmative responses to a brief set of screening questions. However, that screening tool is based on the assumption that all questions are equally predictive of HIV positivity. The other two screening tools based on logistic regression clearly demonstrate violation of that assumption. Therefore, it is not surprising that screening tools based on logistic regression outperformed tools where all questions were equally weighted. However, implementation of screening tools based on the exact logistic method would require use of a spreadsheet or another electronic application to compute screening scores and determine need for HIV testing, which may be challenging to implement at scale in low-resource settings. Fortunately, that limitation is easily overcome by rescaling the estimated coefficients to a practical range and then rounding the rescaled values to whole numbers.

**Fig. 2:**
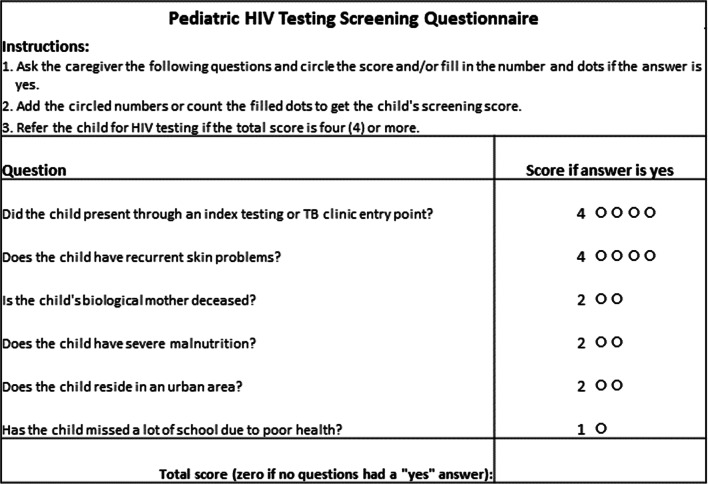
Example pediatric screening tool based on rounded rescaled logistic model coefficients

Rounding and rescaling our logistic coefficients to take values of 1–4 recovers the simplicity of the Z-HRST approach and removes the necessity for electronic applications for implementation. Implementation requires only that clinicians add those values corresponding to affirmative responses to screening questions, and the requirement for addition can be replaced with counting dots (Fig. 2). An HIV risk screening tool based on rescaled logistic regression would allow for simple paper-based implementation by frontline healthcare workers, like the Z-HRST tool, yet with better performance. The rescaled logistic 6-question tool in Ethiopia has the same number of questions, a higher sensitivity (95.0%), and comparable specificity (61.3%) compared to the recently validated pediatric and adolescent risk screening tool in Uganda (sensitivity, 88.1% and specificity, 69.0%, in OPD settings) [39].

Screening tools proposed by studies in Zimbabwe and Malawi for OPD and inpatient settings used clinical screening items to determine eligibility for testing [40–42]. Most of the questions included in the screening tools in this study were similar, including orphan status, skin problems, and poor health. However, while the studies in Zimbabwe and Malawi found that presence of one of the screening items was enough for a positive screen, this study found that a combination of predictors was needed for an optimal screening tool. The use of a combination of factors to determine eligibility for HIV testing may be of higher importance for low-prevalence settings like Ethiopia, to achieve reasonable sensitivity and specificity. A South African study that evaluated the Integrated Management of Childhood Illnesses (IMCI) HIV screening algorithm also showed that a combination of symptoms to determine eligibility for testing could be effective in identifying CLHIV with sensitivity and specificity (71% and 88%, respectively) [43]. The IMCI screening tool was limited for children less than five years of age, while the tools in this study can also be used for older children. However, children who are not attending school at all at the time of screening using our tool would also be less likely to meet the screening criteria compared to older children who are already at school.

While some experts recommend a primary focus on maternal HIV status and exposure screening [44], only 27.0% of newly diagnosed CLHIV reported having a biological relative with HIV in a risk screening validation study from Tanzania [39]. A study in Cameroon, another low HIV prevalence country, found that symptom-based HIV testing combined with index testing maintained the overall number of newly identified CLHIV and improved testing efficiency [45].

Another important feature of the risk screening tools in our study is inclusion of place of residence (urban vs. rural) as a screening item, which could have profound importance in settings such as Ethiopia, which has highly varying HIV prevalence between rural and urban areas. For facilities in rural Ethiopia, where HIV prevalence is lower, implementing this screening algorithm could markedly reduce the number of unnecessary tests while still identifying 95% of CLHIV. Since the elements in the tools are often already routinely asked or evaluated by health care providers, implementation of risk screening algorithms would represent a marginal increase in health worker effort.

Strengths of this study include large sample size from a mix of hospitals and health centers in a low-prevalence country. Data completeness for sociodemographic and clinical data was very high (100%). The study was conducted in multiple service points, which could make it applicable for children presenting to various facility-based pediatric entry points. However, this study has some limitations. This study includes risk screening questions from the literature at the time the study was conducted in 2018, omitting screening questions from more recent studies [39, 42, 45]. The screening tools proposed in this study may not be applicable to the national program and other settings due to the study design. Validation at various pediatric testing entry points, including OPD, will be needed before use of the proposed screening tools at scale and re-validating periodically may be required with changes in the epidemiology of HIV among children.

## Conclusion

The screening tools developed in this study could significantly improve HIV testing efficiency among children presenting to lower yield high-risk entry points in Ethiopia. This study suggests that screening tools developed using combination of screening items to determine eligibility for HIV testing will be more appropriate in low-prevalence settings. Moreover, screening tools that weigh questions differently, using logistic regression, demonstrated superior performance to tools that weighted all questions equally. We believe that tools developed in this study using logistic regression methods will help to systematize pediatric HIV testing practices in Ethiopia and provide a model for other low-prevalence countries to develop targeted testing approaches utilizing country-specific data. Moreover, feasibility, acceptance, and cost effectiveness of the screening tools described in this study needs to be evaluated in the future..

## Data Availability

The datasets generated and/or analyzed during the current study are not publicly available (since the host institution for the research to makes data available only upon request) but are available from the corresponding author on reasonable request.

## Abbreviations

ART: Antiretroviral treatment
CLHIV: Children living with HIV
WHO: World Health Organization
OPD: Outpatient department
NNT: Number needed to test to identify a single new CLHIV
EPHIA: Ethiopian Population-based HIV Impact Assessment
OVC: Orphaned and vulnerable children
PEPFAR: President’s Emergency Plan for AIDS Relief
TB: Tuberculosis
ODK: Open Data Kit
AIC: Akaike information criterion
ROC: Receiver operating characteristic
AUC: Area under the curve
CDC: Centers for Disease Control and Prevention
EPHI: Ethiopian Public Health Institute
IMCI: Integrated Management of Childhood Illnesses
Z-HRST: Zimbabwe HIV Risk Screening Tool

## References

[1] Joint United Nations Programme on HIV/AIDS. Global HIV AIDS Statistics factsheet 2021. https://www.unaids.org/en/resources/fact-sheet. Accessed 18 Aug 2021.

[2] Newell ML, Coovadia H, Cortina-Borja M, Rollins N, Gaillard P, Dabis F (2004). Mortality of infected and uninfected infants born to HIV-infected mothers in Africa: a pooled analysis. Lancet.

[3] Desmonde S, Coffie P, Aka E, Amani-Bosse C, Messou E, Dabis F (2011). Severe morbidity, and mortality in untreated HIV-infected children in a paediatric care programme in Abidjan, Côte d'Ivoire, 2004–2009. BMC Infect Dis.

[4] Marston M, Becquet R, Zaba B, Moulton L, Gray G, Coovadia H (2011). Net survival of perinatally and postnatally HIV-infected children: a pooled analysis of individual data from sub-Saharan Africa. Int J Epidemiol.

[5] World Health Organization. Point-of-care tests for diagnosing HIV infection among children younger than 18 months: 2020. https://www.who.int/publications/i/item/point-of-care-tests-for-diagnosing-hiv-infection-among-children-younger-than-18-months. Accessed 14 Jun 14, 2021.

[6] Kranzer K, Meghji J, Bandason T, Dauya E, Mungofa S, Busza J (2014). Barriers to provider-initiated testing and counselling for children in a high HIV prevalence setting: a mixed methods study. PLoS Med.

[7] World health Organization. Policy requirements for HIV testing of infants and young children in health facilities: 2010. http://apps.who.int/iris/bitstream/10665/44276/1/9789241599092_eng.pdf. Accessed 1 May 2020.

[8] World health Organization. Consolidated guidelines on the use of antiretroviral drugs for treating and preventing HIV infection: recommendations for a public health approach: 2013. http://www.who.int/hiv/pub/arv/arv-2016/en/. Accessed 1 May 2020. 24716260

[9] World health Organization. Consolidated guidelines on HIV testing services: 5Cs: consent, confidentiality, counselling, correct results, and connection: 2015. https://apps.who.int/iris/handle/10665/179870. Accessed 1 May 2020. 26378328

[10] Consolidated guidelines on HIV prevention, testing, treatment, service delivery and monitoring: Recommendations for a public health approach. WHO; 2021 Jul. Report No.: ISBN: 978-92-4-003159-3. https://www.who.int/publications/i/item/9789240031593. 34370423

[11] Ferrand RA, Meghji J, Kidia K, Dauya E, Bandason T, Mujuru H (2016). The effectiveness of routine opt-out HIV testing for children in Harare, Zimbabwe. J Acquir Immune Defic Syndr.

[12] Rollins N, Mzolo S, Moodley T, Esterhuizen T, Rooyen H (2009). Universal HIV testing of infants at immunization clinics: an acceptable and feasible approach for early infant diagnosis in high HIV prevalence settings. AIDS.

[13] Kiyaga C, Urick B, Fong Y, Okiira C, Nabukeera- Barungi N, Nansera D (2018). Where have all the children gone? High HIV prevalence in infants attending nutrition and inpatient settings. J Int AIDS Soc.

[14] Ahmed S, Sabelli R, Simon K, Rosenberg N, Kavuta E, Harawa M (2017). Index case finding facilitates identification and linkage to care of children and young persons living with HIV/AIDS in Malawi. Trop Med Int Health.

[15] McCollum ED, Preidis GA, Golitko CL, Siwande L, Mwansambo C, Kazembe P (2011). Routine inpatient human immunodeficiency virus testing system increases access to pediatric human immunodeficiency virus care in sub-Saharan Africa. Pediatr Infect Dis J.

[16] Kidman R, Anglewicz P (2017). Why are orphaned adolescents more likely to be HIV positive? Distinguishing between maternal and sexual HIV transmission in a meta-analysis of 17 national datasets in Africa. J Adolesc Health.

[17] Central Statistics Agency Ethiopia. 2007 Population and Housing Census of Ethiopia: Statistical Report:2012. https://www.bing.com/search?q=https%3A%2F%2Funstats.un.org%2Funsd%2Fcensuskb20%2FAttachment&form=IPRV10. Accessed 14 Jun 2021.

[18] Ethiopian Public Health Institute. Ethiopia Population-based HIV impact assessment 2017–2018 final report. https://phia.icap.columbia.edu/wp-content/uploads/2020/11/EPHIA_Report_280820_High-Res.pdf. Accessed 21 Jun 2021.

[19] Joint United Nations Programme on HIV/AIDS. Global data on HIV epidemiology and response. https://aidsinfo.unaids.org/. Accessed 23 August 2021.

[20] Federal Democratic Republic of Ethiopia Ministry of Health (FMOH). National Consolidated Guidelines for Comprehensive HIV Prevention, Care and Treatment. August 2018.

[21] Clemens SL, Macneal KD, Alons CL, Cohn JE (2020). Screening algorithms to reduce burden of pediatric HIV testing: a systematic review and meta-analysis. Pediatr Infect Dis J..

[22] Central Statistics Agency Ethiopia. Ethiopia Demographic and health survey. 2016. https://dhsprogram.com/pubs/pdf/FR328/FR328.pdf. Accessed 15 Jun 2021.

[23] Hrapcak S, Bekele A, Ahmed J, Ayalew J, Gutreuter S, Kumssa H (2021). Finding children living with HIV in low-prevalence countries: HIV prevalence and testing yield from 5 entry points in Ethiopia. Pediatr Infect Dis.

[24] The Federal Democratic Republic of Ethiopia Ministry of Science and Technology. National Health Research Ethics Review Guideline, Fifth Edition, 2013.

[25] Click ES, Feleke B, Pevzner E, Fantu R, Gadisa T, Assefa D (2012). Evaluation of integrated registers for tuberculosis and HIV surveillance in children in Ethiopia, 2007–2009. Int J Tuberc Lung Dis.

[26] The Federal Democratic Republic of Ethiopia Ministry of Health (FMOH). Guidelines for the management of acute malnutrition, FMOH. 2016

[27] Bandason T, McHugh G, Dauya E, Mungofa S, Munyati S, Weiiss H (2016). Validation of a screening tool to identify older children living with HIV in primary care facilities in high HIV prevalence settings. AIDS.

[28] Zinyemba T, Milena Pavlova M, Groot W (2019). Effects of HIV/AIDS on children's educational attainment: a systematic literature review. J Econ Surv.

[29] Hastie T, Tibshirani R, Friedman J (2009). The elements of statistical learning.

[30] Park MY, Hastie T (2007). *L*1-regularization path algorithm for generalized linear models. J R Stat Soc Ser B.

[31] Kim J-H (2009). Estimating classification error rate: Repeated cross-validation, repeated hold-out and bootstrap. Comput Stat Data Anal.

[32] Rodríguez JD, Pérez A, Lozano JA (2010). Sensitivity analysis of *k*-fold cross validation in prediction error estimation. IEEE Trans Pattern Anal Mach Intell.

[33] screenr: An R package to enable screening in/out subjects who are likely to test positive/negative, respectively. https://github.com/sgutreuter/screenr. Accessed 22 April 2022.

[34] R Core Team. R: a language and environment for statistical computing Vienna, Austria: R Foundation for Statistical Computing; 2020. http://www.R-project.org/.

[35] Robin X, Turck N, Hainard A (2011). pROC: an open-source package for R and S+ to analyze and compare ROC curves. BMC Bioinf.

[36] United Nations International Children's Emergency Fund. Elimination of Mother to child transmission: fast facts. https://data.unicef.org/topic/hivaids/emtct/. Accessed 29 July 2021.

[37] World Health Organization. Family-based index case testing to identify children with HIV: 2019. https://www.who.int/publications/i/item/WHO-CDS-HIV-19.24. Accessed 17 July 2020.

[38] Bandyopadhyay A, Bhattacharyya S, Banerjee A (2009). Clinicoepidemiological scoring system for early diagnosis of pediatric HIV. Indian Pediatr.

[39] Katureebe C, Ashburn K, Machekano R, Gill MM, Gross J, Kazooba P (2021). Developing and validating an effective pediatric and adolescent HIV testing eligibility screening tool for high-volume entry points in Uganda. J Acquir Immune Defic Syndr.

[40] Bandason T, Dauya E, Dakshina S, McHugh G, Chonzi P, Munyati S (2018). Screening tool to identify adolescents living with HIV in a community setting in Zimbabwe: a validation study. PLoS ONE.

[41] Moucheraud C, Chasweka D, Nyirenda M, Schooley A, Dovel K, Hoffman R (2018). Simple screening tool to help identify high-risk children for targeted HIV testing in Malawian inpatient wards. J Acquir Immune Defic Syndr.

[42] Antelman G, Gill MM, Jahanpour O, van de Ven R, Khabuka C, Barankana A (2021). Balancing HIV testing efficiency with HIV case-identification among children and adolescents (2–19 years) using an HIV risk screening approach in Tanzania. PLoS ONE.

[43] Horwood C, Liebeschuetz S, Blaauw D, Cassol S, Qazi S (2003). Diagnosis of paediatric HIV infection in a primary health care setting with a clinical algorithm. Bull World Health Organ.

[44] Ahmed S, Cox C, Abrams E (2020). Commentary on “symptom-based screening is not the solution to improve pediatric HIV testing”. Pediatr Infect Dis J.

[45] Yumo HA, Ajeh RA, Beissner M, Ndenkeh JN, Sieleunou I, Jordan MR (2019). Effectiveness of symptom-based diagnostic HIV testing versus targeted and blanket provider-initiated testing and counseling among children and adolescents in Cameroon. PLoS ONE.

